# C9orf72 associates with inactive Rag GTPases and regulates mTORC1‐mediated autophagosomal and lysosomal biogenesis

**DOI:** 10.1111/acel.13126

**Published:** 2020-02-25

**Authors:** Mingmei Wang, Hongfeng Wang, Zhouteng Tao, Qin Xia, Zongbing Hao, Jochen H. M. Prehn, Xuechu Zhen, Guanghui Wang, Zheng Ying

**Affiliations:** ^1^ Jiangsu Key Laboratory of Neuropsychiatric Diseases and College of Pharmaceutical Sciences Soochow University Suzhou China; ^2^ School of Pharmacy Key Laboratory of Molecular Pharmacology and Drug Evaluation (Yantai University) Ministry of Education Yantai University Yantai China; ^3^ Department of Physiology & Medical Physics and FUTURE‐NEURO Research Centre Royal College of Surgeons in Ireland Dublin 2 Ireland; ^4^ Jiangsu Key Laboratory of Preventive and Translational Medicine for Geriatric Diseases College of Pharmaceutical Sciences Soochow University Suzhou China

**Keywords:** autophagy, mTOR complex 1, neurodegenerative diseases, Rag GTPases, transcription factor EB

## Abstract

GGGGCC repeat expansion in *C9orf72* is the most common genetic cause in both frontotemporal dementia (FTD) and amyotrophic lateral sclerosis (ALS), two neurodegenerative disorders in association with aging. Bidirectional repeat expansions in the noncoding region of *C9orf72* have been shown to produce dipeptide repeat (DPR) proteins through repeat‐associated non‐ATG (RAN) translation and to reduce the expression level of the *C9orf72* gene product, C9orf72 protein. Mechanisms underlying *C9orf72*‐linked neurodegeneration include expanded RNA repeat gain of function, DPR toxicity, and C9orf72 protein loss of function. In the current study, we focus on the cellular function of C9orf72 protein. We report that C9orf72 can regulate lysosomal biogenesis and autophagy at the transcriptional level. We show that loss of C9orf72 leads to striking accumulation of lysosomes, autophagosomes, and autolysosomes in cells, which is associated with suppressed mTORC1 activity and enhanced nuclear translocation of MiT/TFE family members MITF, TFE3, and TFEB, three master regulators of lysosomal biogenesis and autophagy. We demonstrate that the DENN domain of C9orf72 specifically binds to inactive Rag GTPases, but not active Rag GTPases, thereby affecting the function of Rag/raptor/mTOR complex and mTORC1 activity. Furthermore, active Rag GTPases, but not inactive Rag GTPases or raptor rescued the impaired activity and lysosomal localization of mTORC1 in C9orf72‐deficient cells. Taken together, the present study highlights a key role of C9orf72 in lysosomal and autophagosomal regulation, and demonstrates that Rag GTPases and mTORC1 are involved in C9orf72‐mediated autophagy.

## Graphical abstract

Pathogenic mutation in C9orf72 is the most common cause of frontotemporal dementia and amyotrophic lateral sclerosis. Loss of C9orf72 protein will decrease the activity of Rag GTPases and mTOR lysosomal localization. C9orf72 mediates autophagosomal and lysosomal biogenesis through Rag GTPase‐mTORC1‐MITF/TFE3/TFEB signaling axis.

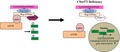

## 1 INTRODUCTION

Frontotemporal dementia (FTD) and amyotrophic lateral sclerosis (ALS) are aging‐related neurodegenerative disease sharing certain susceptibility genes and pathological features (Ling, Polymenidou, & Cleveland, 2013; Niccoli, Partridge, & Isaacs, 2017). Expansion of GGGGCC hexanucleotide repeat in the noncoding region of *C9orf72* is the most common cause of both diseases (DeJesus‐Hernandez et al., 2011; Renton et al., 2011). Interestingly, recent studies showed that *C9orf72* hexanucleotide repeat expansions also exist in Alzheimer's disease and other neurodegenerative disorders (Majounie et al., 2012), providing evidence that the etiology of various aging‐related neurodegenerative diseases involves common molecular mechanism associated with *C9orf72*. Expanded *C9orf72* hexanucleotide RNA repeats can form RNA foci and sequester RNA‐binding proteins and therefore may cause neurodegeneration through RNA toxicity. In addition, the RNA repeats can be translated into five different types of dipeptide repeat (DPR) proteins through a unconventional translation mechanism named repeat‐associated non‐ATG (RAN) translation (Ash et al., 2013; Mori et al., 2013; Zu et al., 2013). The hexanucleotide repeat expansions also block the transcription of the *C9orf72* coding gene product (DeJesus‐Hernandez et al., 2011; Renton et al., 2011), due to epigenetic regulation (Belzil et al., 2013; Xi et al., 2013) and abortive transcription (Haeusler et al., 2014). Therefore, both gain‐ and loss‐of‐function mechanisms have been proposed for *C9orf72*‐linked ALS/FTD (Moens, Partridge, & Isaacs, 2017). For the protein loss‐of‐function mechanism, it has been reported that the RNA transcript and protein levels of C9orf72 are reduced in *C9orf72*‐linked ALS/FTD, possibly also resulting from the expansion of the hexanucleotide repeat (DeJesus‐Hernandez et al., 2011; Haeusler et al., 2014; Renton et al., 2011). Despite lack of evidence in mouse models, several studies showed that loss of C9orf72 in worm and zebrafish models induces motor neuron degeneration and motor deficits (Ciura et al., 2013; Therrien, Rouleau, Dion, & Parker, 2013), suggesting loss of C9orf72 function may partially contribute to disease pathogenesis of *C9orf72*‐linked ALS/FTD (Moens et al., 2017).

The protein function of *C9orf72*‐encoded C9orf72 remains largely unknown. Bioinformatic analysis suggested that C9orf72, similar to folliculin, FNIP1/2, and SMCR8, structurally belonged to the DENN‐like protein family (Zhang, Iyer, He, & Aravind, 2012). DENN‐like proteins play important roles in membrane trafficking, through their actions as RAB GTPase GDP/GTP exchange factor (RAB GEF) (Zhang et al., 2012). Indeed, recent studies revealed that C9orf72 forms a complex with SMCR8/WDR41 and associates with RAB1, RAB5, RAB7, RAB8A, RAB11, and RAB39B GTPases, and hence could play a role in the regulation of endosomal trafficking and autophagy at multiple steps (Amick, Roczniak‐Ferguson, & Ferguson, 2016; Corrionero & Horvitz, 2018; Farg et al., 2014; Sellier et al., 2016; Shi et al., 2018; Sullivan et al., 2016; Ugolino et al., 2016; Webster et al., 2016; Yang et al., 2016). Interestingly, recent studies showed that the level of LC3‐II, which reflects the number of autophagosomes in cells, was up‐regulated in C9orf72‐deficient tissue culture cells (Farg et al., 2014; Ugolino et al., 2016; Yang et al., 2016). In support of this, *C9orf72‐*deficient mice also showed increased levels of autophagic components, such as LC3 (O'Rourke et al., 2016; Sullivan et al., 2016; Ugolino et al., 2016).

Autophagy is a cellular protein and organelle quality control system which degrades protein aggregates, damaged organelles, and pathogens through lysosomes (Dikic & Elazar, 2018). It is well known that autophagy plays important roles in disease progress of neurodegeneration, and dysregulation of autophagy has been broadly implicated in the disease pathogenesis of neurodegenerative diseases including ALS (Nguyen, Thombre, & Wang, 2018). Recently, the microphthalmia/transcription factor E (MiT/TFE) family of helix‐loop‐helix/leucine‐zipper transcription factors, including microphthalmia‐associated transcription factor (MITF) transcription factor EB (TFEB), TFE3, and TFEC, has been recently identified as master regulators of lysosomal biogenesis and autophagy by regulating the global gene expression of the coordinated lysosomal expression and regulation (CLEAR) gene network (Puertollano, Ferguson, Brugarolas, & Ballabio, 2018). The nucleocytoplasmic shuttling of TFEB is mainly regulated by mTOR complex 1 (mTORC1), an important protein complex connected with Rag GTPases and Ragulator on the lysosome membrane (Martina, Chen, Gucek, & Puertollano, 2012; Puertollano et al., 2018; Roczniak‐Ferguson et al., 2012).

In the present study, we report that in C9orf72‐deficient cells, the activity and lysosomal localization of the mTORC1 were strikingly suppressed. We show that C9orf72 can specifically associate with inactive Rag GTPases, but not active Rag GTPases, through its DENN domain. Moreover, we find that the C9orf72‐Rag‐mTORC1 signal axis controls global gene expression of autophagosomal and lysosomal genes by targeting multiple transcriptional factors including MITF, TFE3, and TFEB.

## 2 RESULTS

### 2.1 C9orf72‐deficient cells display abnormal accumulation of autophagosomes and lysosomes associated with impaired mTORC1 activity and increased TFEB nuclear translocation

**Figure 1:**
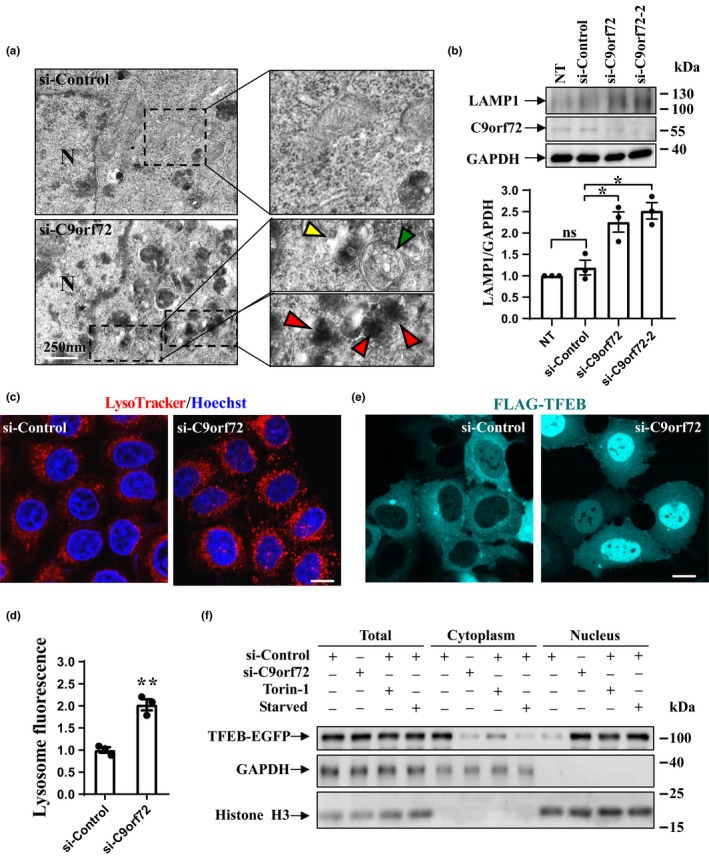
Effect of C9orf72 on autophagic components. (a) HeLa cells were transfected with either negative control siRNA or C9orf72 siRNA. Double‐membrane autophagosomes are indicated with green arrowhead, and autolysosomes are indicated with red arrowhead. Dotted boxes are magnified on the right side. Scale bar, 250 nm. (b) HEK 293 cells were transfected with the indicated siRNAs or treated with RNAi MAX reagent only (no transfection, NT). Cell lysates were subjected to immunoblot analysis using antibodies against LAMP1, C9orf72, and GAPDH. The relative densities of LAMP1 to GAPDH are shown on the lower side. Data from three independent experiments are represented as mean ± *SEM*, ns, not significantly different; * *p* < .05, one‐way ANOVA. (c) HEK 293 cells were transfected with either control or C9orf72 siRNA. Seventy‐two hours later, the cells were firstly stained with LysoTracker (red) and then fixed. Hoechst (blue) was used for nuclear staining. Scale bar, 5 µm. (d) Quantifications of lysosome fluorescence intensity are shown as mean ± *SEM*, ** *, p* < .01, one‐way ANOVA. (e) HEK 293 cells were transfected with the indicated siRNAs for 48 hr. Then, the cells were re‐transfected with FLAG‐TFEB. Twenty‐four hours later, the cells were subjected to immunofluorescent assay with FLAG antibody and visualized using confocal microscopy. Scale bar, 5 µm. (f) HEK 293 cells were transfected with indicated siRNAs for 80 hr, followed by second transfection with TFEB‐EGFP. Then, the cells were treated with Torin‐1 (250 nM) or Earle's balanced salt solution (starvation) for 1 hr. Cell lysates were separated into cytoplasm and nuclear fractions, and the fractions were subjected to immunoblot analysis with GFP, GAPDH, and Histone H3 antibodies

Because accumulation of autophagic vesicles is a common pathological feature of neurodegenerative disorders such as ALS (Ling et al., 2013), we examined the autophagic structures in C9orf72‐deficient cells using electron microscopy (EM). Autolysosomes were identified on the basis of typical high electron density and the presence of multiple luminal vesicles. Meanwhile, autophagosomes were identified on the basis of double‐membrane structures containing internal contents. EM analysis showed strikingly increased numbers of autophagosomes and autolysosomes as a consequence of C9orf72 knockdown (Figure 1a). We next performed C9orf72 knockdown and immunoblot experiments, and we found that the protein level of LAMP1, a lysosomal marker protein, was increased in C9orf72‐deficient cells using different siRNAs targeting C9orf72 (Figure 1b), indicating these observations were not caused by off‐target effects. For subsequent studies, we focused on one siRNA (named si‐C9orf72) for C9orf72 knockdown experiments, which produced an efficient silencing of C9orf72. LysoTracker is a fluorescent dye used to monitor lysosomal biogenesis (Xia et al., 2016). We observed increased signal of LysoTracker staining (Figure 1c), indicating lysosomal biogenesis was enhanced in C9orf72‐deficient cells.

**Figure 2:**
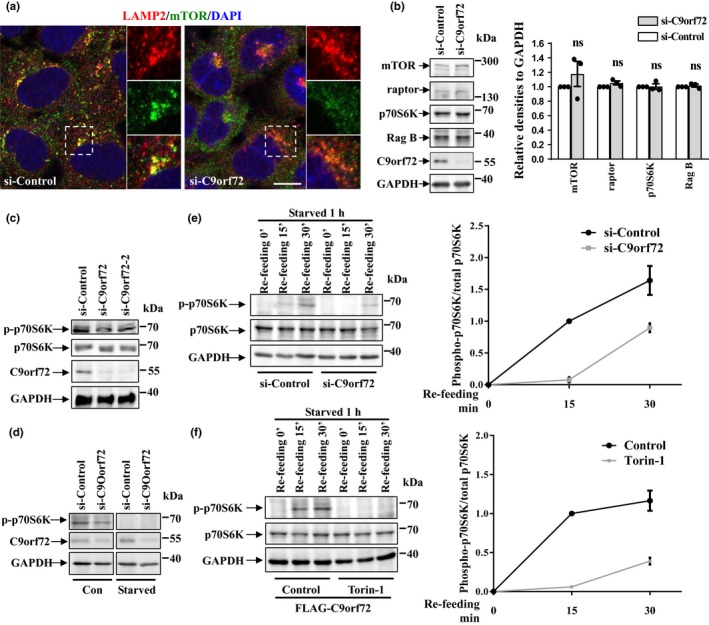
Effect of C9orf72 on mTOR localization and mTORC1 activity. (a) HEK 293 cells were transfected with either control or C9orf72 siRNA for 72 hr, and then, the cells were subjected to immunofluorescence assay by antibody against LAMP2 (red) and mTOR (green). DAPI (blue) was used for nuclear staining. Scale bar, 5 µm. (b) HEK 293 cells were transfected as in A. After 72 hr, the cells were lysed and lysates were subjected to immunoblot assay using indicated antibodies. Statistical analysis from three independent experiments was presented as means ± *SEM*, ns, not significantly different; one‐way ANOVA. (c) HEK 293 cells were transfected with the indicated siRNAs for 72 hr. Then, the cell lysates were subjected to immunoblot analysis. (d) HEK 293 cells were transfected with the indicated siRNAs for 72 hr. Then, the cells were then incubated with Earle's balanced salt solution for 1 hr. The cell lysates were then subjected to immunoblot analysis. (e) HEK 293 cells were transfected with the indicated siRNAs for 72 hr. Then, the cells were incubated with EBSS for 1 hr and re‐fed with full medium for 15 or 30 min. The cell lysates were subjected to immunoblot analysis with indicated antibodies. The statistical analysis of relative densities is shown on the right side as means ± *SD*. (f) HEK 293 cells were transfected with FLAG‐tagged C9orf72. Twenty‐four hours later, the cells were incubated with EBSS for 1 hr or re‐feeding with amino acids for the indicated time, with or without Torin‐1 (250 nM) treatment. The statistical analysis of relative densities is shown on the right side as means ± *SD*

Given that lysosome biogenesis was increased in C9orf72‐deficient cells (Figure 1a,b), we decided to examine whether C9orf72 could regulate TFEB. As indicated by immunofluorescence and biochemical fractionation assays, TFEB normally localized in the cytosol but translocated into the nucleus when we silenced C9orf72 in the cells (Figure 1e,f). Similar effects were seen in cells subjected to starvation condition or treated with Torin‐1 which served as positive controls (Figure 1f) (Martina et al., 2012; Roczniak‐Ferguson et al., 2012). We next checked the cellular localization of mTOR and activity of mTORC1, the upstream regulator of TFEB. We found that lysosome‐like localization of mTOR, which reflects the activity of mTOR, was abolished when C9orf72 was depleted (Figure 2a, LAMP2 indicates lysosome). Although the protein levels of the components in Ragulator‐Rag‐mTORC1 complex, including mTOR, raptor, and Rag B, were not affected (Figure 2b), phosphorylated p70S6K (a well‐recognized mTORC1 substrate that can be used as a reporter of mTORC1 activity) at threonine 389 was significantly reduced (Figure 2c). Phosphorylation of p70S6K was diminished in starved cells (Figure 2d) and was recovered during nutrition re‐feeding (Figure 2e). We found that knockdown of C9orf72 significantly delayed the recovery of p70S6K phosphorylation during re‐feeding (Figure 2e). Importantly, the mTOR inhibitor Torin‐1 strongly blocked the recovery of p70S6K phosphorylation in C9orf72‐expressing cells, indicating that C9orf72 regulates mTORC1 signaling and functions upstream of mTOR (Figure 2f).

### 2.2 C9orf72 regulates mTORC1 activity, mTOR, and TFEB cellular localization through Rag GTPases

**Figure 3:**
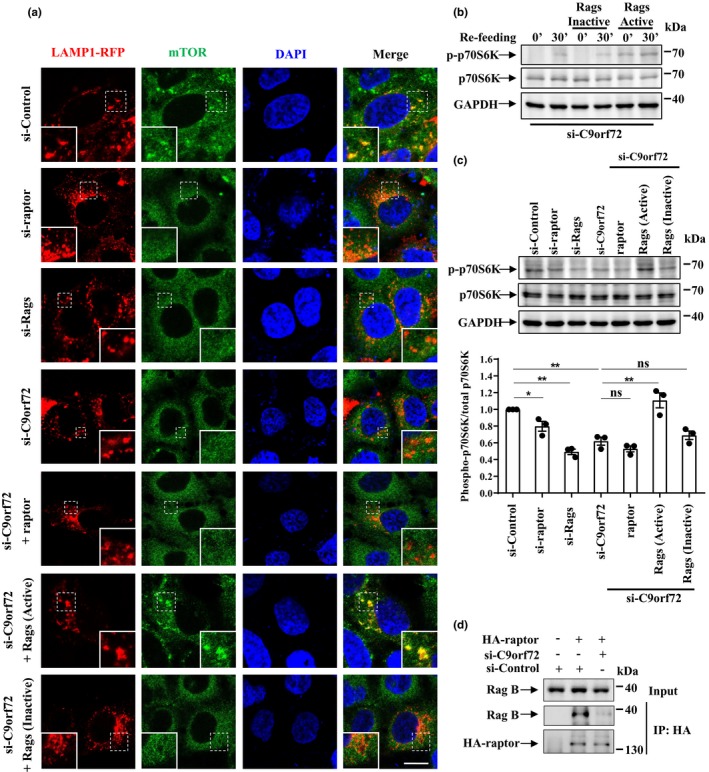
C9orf72 controls the lysosomal localization of mTOR through Rag GTPases. (a) HEK 293 cells were transfected with the indicated siRNAs for 48 hr; then, the cells were re‐transfected with LAMP1‐RFP (red), along with HA‐raptor, HA‐GST‐tagged constitutively active Rag GTPase mutants (Rag A Q66L + Rag C S75L = Rag A^GTP^ + Rag C^GDP^, hereafter referred to as active Rags), or constitutively inactive Rag GTPase mutants (Rag A T21L + Rag C Q120L = Rag A^GDP^ + Rag C^GTP^, hereafter referred to as inactive Rags). The cells were fixed and stained with anti‐mTOR antibody (green) and DAPI (blue). Regions with dotted boxes are magnified in the insets. Scale bar, 5 µm. (b) HEK 293 cells were transfected with constitutively active or inactive Rags for 24 hr. Then, the cells were starved with EBSS for 1 hr and re‐stimulated with amino acids for 30 min. The cell lysates were then subjected to immunoblot analysis. (c) HEK 293 cells were transfected as described in A. The cell lysates were collected for immunoblot analysis using antibody against phosphorylated p70S6K (T389), p70S6K, or GAPDH. The quantitative data from three independent experiments were shown as means ± *SEM*, ns, not significantly different; * *p* < .05; ** *p* < .01, one‐way ANOVA. (d) HEK 293 T cells were transfected with indicated siRNAs and HA‐raptor for 72 hr. Then, the supernatant of cell lysates was subjected to immunoprecipitation assay with HA antibody. Note that we used Rag B antibody to detect endogenous Rag GTPase since Rag B is very similar to Rag A

Previous studies showed that the cellular localization of mTOR and the activity of mTORC1 can be regulated by raptor or Rag GTPases, the effector in Ragulator‐Rag‐mTORC1 complex (Sancak et al., 2010). We next asked whether C9orf72 could affect mTORC1 by regulating raptor or Rag GTPases. In control cells, mTOR colocalized with LAMP1, indicating mTOR could localize on the lysosome membrane to maintain its activity (Figure 3a, upper panel). In contrast, the lysosomal localization of mTOR was reduced in C9orf72‐deficient cells, as well as in raptor or Rag GTPases‐deficient cells (Figure 3a). The knockdown effects of raptor or Rag GTPases are shown in Figure S1. The Rag family of GTPases functions as heterodimer proteins in which the active form contains GTP‐bound Rag A or Rag B (very similar to Rag A) and GDP‐bound Rag C or Rag D (very similar to Rag C), and the inactive form contains GDP‐bound Rag A or Rag B and GTP‐bound Rag C or Rag D). Based on this, we chose to study Rag heterodimers containing A and C, since they have the similar biological functions as the heterodimers containing B and D. Interestingly, we found that the lysosomal localization of mTOR was restored in C9orf72‐deficient cells expressing active Rag GTPase heterodimers (Rag A^GTP^/Rag C^GDP^), while expression of raptor or inactive Rag GTPase heterodimers (Rag A^GDP^/Rag C^GTP^) failed to rescue mTOR lysosomal localization (Figure 3a, lower panels). Consistently, we found that the activity of mTORC1, indicated by phosphorylated p70S6K, was also decreased by C9orf72, raptor, or Rag GTPase knockdown and was rescued by active Rag GTPases in C9orf72‐deficient cells, whereas it was not rescued by raptor or inactive Rag GTPases (Figure 3b,c). Moreover, our results showed that knockdown of C9orf72 could dramatically decrease the association between endogenous Rag B and raptor, suggesting that the activity of endogenous Rag GTPases was decreased in C9orf72‐deficient cells (Figure 3d).

**Figure 4:**
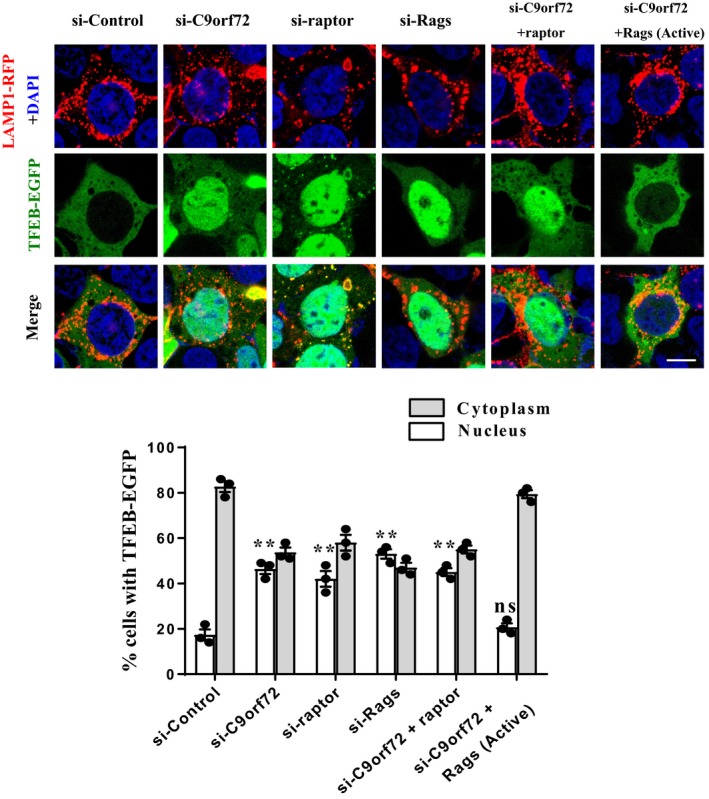
C9orf72 controls the nucleocytoplasmic shuttling of TFEB through Rag GTPases. HEK 293 cells were transfected with the indicated siRNAs. After 48 hr, the cells were transfected with TFEB‐EGFP (green) and LAMP1‐RFP (red), along with HA‐raptor or HA‐GST‐Rags for another 24 hr. The cells were fixed and stained with DAPI (blue). Scale bar, 5 µm. The quantification data of TFEB nucleus and cytoplasm localization are shown below. Statistical significance of percentage of TFEB‐EGFP nuclear localization was assessed to si‐Control group. Data were shown as means ± *SEM*, ns, not significantly different; ** *p* < .01, one‐way ANOVA

In consistence with previous studies (Martina et al., 2012; Roczniak‐Ferguson et al., 2012), we found that TFEB normally localized in the cytoplasm, whereas it translocated to the nucleus in raptor or Rag GTPase‐depleted cells (Figure 4), indicating mTORC1 activity was repressed in these cells. To elucidate the mechanism underlying C9orf72‐mediated TFEB nucleocytoplasmic shuttling, we performed the rescue assay as in Figure 3. Our results showed that silencing of C9orf72 resulted in TFEB nuclear translocation in both neuronal and non‐neuronal cells (Figure 4 and Figure S2a). TFEB was able to shuttle back to the cytoplasm after expression of active Rag GTPases, but not after expression of raptor or inactive Rag GTPases in C9orf72‐deficient cells (Figure 4 and Figure S2b–d). Taken together, our data suggest a critical role of Rag GTPases in C9orf72‐mediated cellular localization of mTORC1 and TFEB.

### 2.3 C9orf72 interacts with inactive Rag GTPases

**Figure 5:**
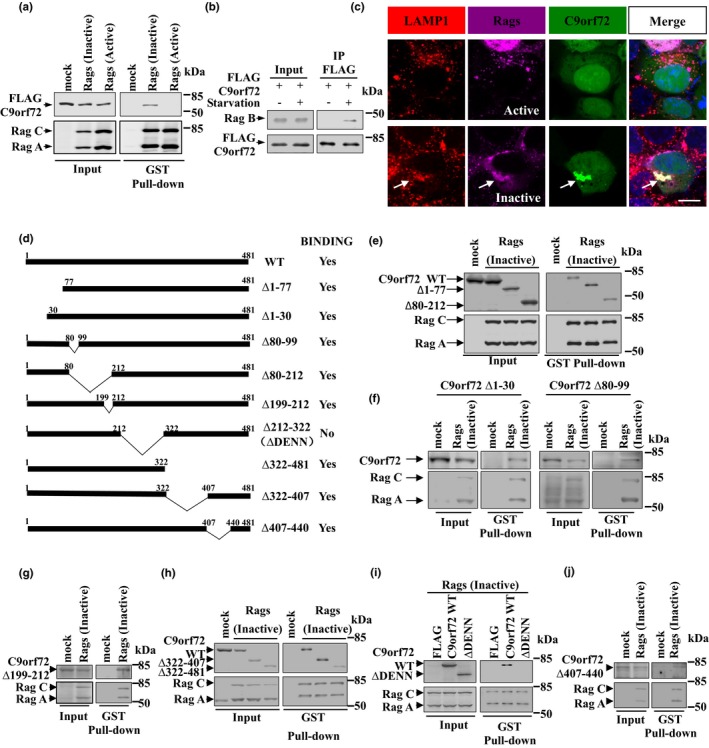
C9orf72 associates with the inactive form of Rag GTPases via DENN domain. (a) HEK 293T cells were co‐transfected with HA‐GST‐tagged active or inactive Rags, along with FLAG‐C9orf72. The “mock” was HA‐tag‐only overexpression which serves as the negative control. Forty‐eight hours later, cells were collected, and the supernatants of the cell lysates were incubated with G4B magnetic beads. Bound proteins were detected using FLAG and HA antibodies. (b) FLAG‐C9orf72 was transfected into HeLa cells. Twenty‐four hours later, the cells were incubated with full medium or EBSS for 1 hr. The supernatants of the cell lysates were immunoprecipitated with antibody against FLAG. Bound proteins were detected using FLAG or Rag B (relative similar to Rag A) antibodies. (c) HEK 293 cells were transfected with HA‐GST‐tagged Rags and C9orf72‐EGFP. Forty‐eight hours later, the cells were fixed and subjected to immunofluorescence staining with HA, LAMP1 antibody (for detecting lysosome) and DAPI. The “Merge” panels contain the signal from DAPI staining. Arrows indicates colocalization. Scale bar, 5 µm. (d) Schematic diagram of C9orf72 constructs used in this study. (e) HEK 293T cells were transfected with mock vector or HA‐GST‐tagged constitutively inactive Rags and FLAG‐tagged full‐length (WT), Δ1–77, or Δ80–212 C9orf72. Forty‐eight hours later, the supernatants of the cell lysates were used in GST pull‐down assay. Bound proteins were detected with HA and FLAG antibodies. (f) HEK 293T cells were transfected with mock vector or HA‐GST‐tagged constitutively inactive Rags and EGFP‐tagged Δ1–30 or Δ80–99 C9orf72. Forty‐eight hours later, the supernatants of the cell lysates were used in GST pull‐down assay. Bound proteins were detected with HA and GFP antibodies. (g) HEK 293T cells were transfected with mock vector or HA‐GST‐tagged constitutively inactive Rags and EGFP‐tagged Δ199–212 C9orf72. Forty‐eight hours later, the supernatants of the cell lysates were used in GST pull‐down assay. Bound proteins were detected with HA and GFP antibodies. (h) HEK 293T cells were transfected with mock vector or HA‐GST‐tagged constitutively inactive Rags and FLAG‐tagged full‐length, Δ322–407, or Δ322–481 C9orf72. Forty‐eight hours later, the supernatants of the cell lysates were used in GST pull‐down assay. Bound proteins were detected with HA and FLAG antibodies. (i) HEK 293T cells were transfected with HA‐GST‐tagged constitutively inactive Rags and FLAG, FLAG‐tagged full‐length or Δ212–322 (ΔDENN) C9orf72. Forty‐eight hours later, the supernatants of the cell lysates were used in GST pull‐down assay. Bound proteins were detected with HA and FLAG antibodies. (j) HEK 293T cells were transfected with mock vector or HA‐GST‐tagged constitutively inactive Rags and EGFP‐tagged Δ407–440 C9orf72. Forty‐eight hours later, the supernatants of the cell lysates were used in GST pull‐down assay. Bound proteins were detected with HA and GFP antibodies

There are two C9orf72 protein isoforms in humans. Since previous studies suggest a strong relationship between the longer isoform and autophagy (Sellier et al., 2016; Sullivan et al., 2016; Yang et al., 2016), we mainly focused on the longer isoform in the present study. We generated two constructs of the longer C9orf72 tagged with EGFP or FLAG (named C9orf72‐EGFP and FLAG‐C9orf72) and performed biochemical and immunofluorescent analyses to test whether there was protein interaction between C9orf72 and Rag GTPases. To explore the potential interaction between C9orf72 and inactive Rag GTPases, we co‐transfected cells with C9orf72 and Rag GTPases and performed GST pull‐down and immunoprecipitation assays. Our results showed that inactive Rag GTPases, but not active Rag GTPases, could interact with C9orf72 (Figure 5a and Figure S3). The interaction between C9orf72 and endogenous Rag protein was observed during starvation, a condition that turns Rag GTPases into the inactive conformation (Figure 5b). C9orf72‐EGFP displayed punctuate structures in the cytoplasm of cells expressing inactive Rag GTPases, and those punctuate structures strongly colocalized with inactive Rag GTPases and LAMP1 (Figure 5c, lower panel). In contrast, C9orf72 did not colocalize with active Rag GTPases (Figure 5c, upper panel). These results suggest that inactive Rag GTPases, but not active Rag GTPases, can associate with C9orf72 and recruit C9orf72 onto the lysosomes.

### 2.4 DENN domain is required for the interaction between C9orf72 and inactive Rag GTPases

To further examine which domain of C9orf72 was necessary for the interaction with inactive Rag GTPases, we generated a series of deletion mutants of C9orf72 (Figure 5d) and tested their interactions with inactive Rag GTPases. We identified that the wild‐type C9orf72, C9orf72 (Δ1–30), C9orf72 (Δ1–77), C9orf72 (Δ80–99), C9orf72 (Δ80–212), C9orf72 (Δ199–212), C9orf72 (Δ322–481), C9orf72 (Δ322–407), and C9orf72 (Δ407–440) bound to inactive Rag GTPases, whereas C9orf72 lacking DENN domain (Δ212–322) did not (Figure 5e‐j). In addition, immunofluorescent analysis showed that wild‐type C9orf72, C9orf72 (Δ1–77), C9orf72 (Δ80–212), and C9orf72 (Δ322–481), but not C9orf72 (ΔDENN/Δ212–322), colocalized with inactive Rag GTPases in cells (Figure S4). In addition, we found that re‐expression of C9orf72 ΔDENN failed to recover the lysosomal biogenesis and TFEB localization in C9orf72‐depleted cells (Figure S5). Taken together, our data suggested that the DENN domain of C9orf72 was critical for the interaction between C9orf72 and inactive Rag GTPases and C9orf72‐mediated TFEB cellular localization.

### 2.5 C9orf72 loss of function results in abnormal regulation of autophagosomal and lysosomal biogenesis through MITF/TFE3/TFEB transcription factors

**Figure 6:**
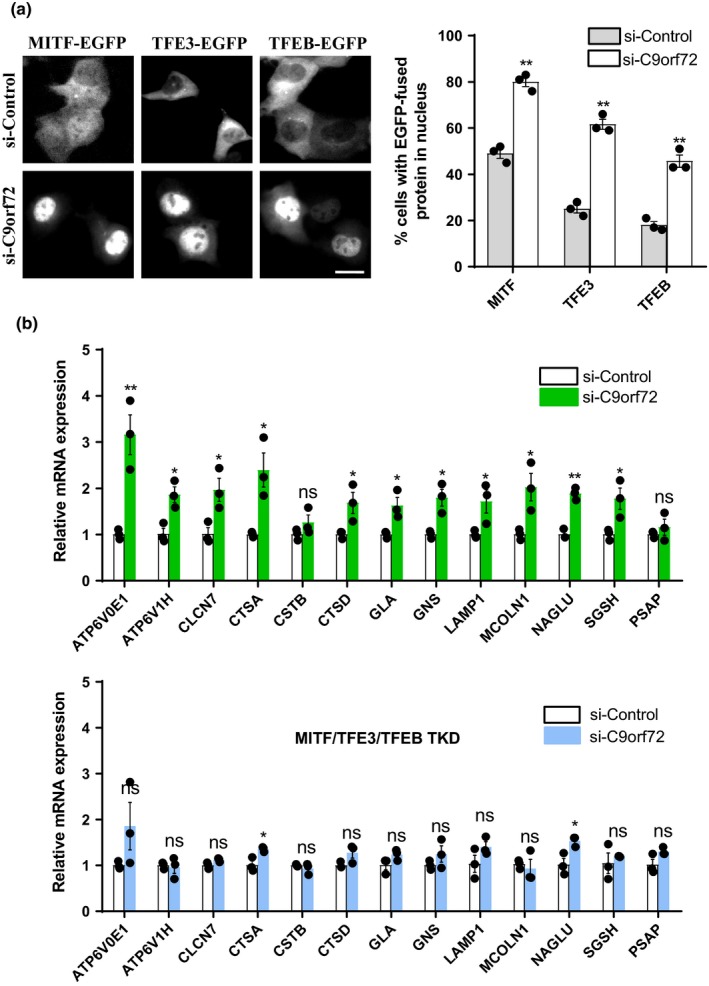
C9orf72 regulates autophagosomal and lysosomal biogenesis by targeting MITF/TFE3/TFEB. (a) HEK 293 cells were transfected with control or C9orf72 siRNAs. Forty‐eight hours later, the cells were re‐transfected with EGFP‐tagged MITF, TFE3, or TFEB. After 24 hr, the EGFP‐fused proteins were visualized using an inverted system microscope. Scale bar, 10 µm. The quantifications of EGFP‐fused protein localization in nucleus are shown on the right side as means ± *SEM*, ** *p* < .01, one‐way ANOVA. (b) HEK 293 cells were transfected with control or C9orf72 siRNAs, with or without MITF/TFE3/TFEB siRNAs. Seventy‐two hours later, the cells were processed for qRT–PCR analysis. The mRNA levels of autophagic and lysosomal genes were quantified and normalized to GAPDH. Data were shown as means ± *SEM*, ns, not significantly different; * *p* < .05; ** *p* < .01, one‐way ANOVA

Similar to the observations made on TFEB nuclear translocation (Figure 1e and Figure 6a), other MiT/TFE family members, including MITF and TFE3, also translocated to the nucleus in C9orf72‐deficient cells (Figure 6a). Since nuclear translocation of these MiT/TFE family proteins could globally enhance the transcription of genes in autophagy and lysosome systems (Puertollano et al., 2018), thereby generating new autophagosomes and lysosomes and increasing autophagic flux, we wondered whether C9orf72 could regulate the biogenesis of autophagosomes and lysosomes through MITF/TFE3/TFEB. We chose to analyze a subset of previously reported MiT/TFE family protein‐targeting genes (Martina et al., 2014; Settembre et al., 2011; Xia et al., 2016), and our results showed that the transcriptional expression levels of many autophagosomal and lysosomal genes, but not the genes associated with ALS/FTD, were up‐regulated in C9orf72‐deficient cells (Figure 6b and Figure S6). Meanwhile, they were not significantly changed in cells lacking MITF, TFE3, and TFEB (Figure 6b), suggesting that C9orf72 controls autophagosomal and lysosomal biogenesis in an MITF/TFE3/TFEB‐dependent manner. We further used a recent developed autophagic flux probe GFP‐LC3‐RFP‐LC3ΔG (Kaizuka et al., 2016) to access autophagic flux in C9orf72‐deficient cells. When expressed in cells, GFP‐LC3‐RFP‐LC3ΔG is cleaved by ATG4 into degradable and quenchable GFP‐LC3 and nondegradable RFP‐LC3ΔG. GFP‐LC3 is degraded by lysosome; meanwhile, RFP‐LC3ΔG stays in the cells as an internal control. As shown in Figure S7, loss of C9orf72 decreased the level of GFP‐LC3‐II (immunoblot. S7a) and the intensity of GFP‐LC3 signal (microscopy, S7b), indicating the overall autophagic flux was increased in C9orf72‐deficient cells. We also found that loss of C9orf72 decreased the protein level of the autophagic substrate p62 (SQSTM1/p62) and increased LC3‐II (Figure S7c,d). In addition, we performed lysosomal pH measurement and found that C9orf72 depletion did not affect lysosomal pH (Figure S7e), indicating that the activation of TFEB related transcription factors was a primary event driven by Rag GTPase‐mTORC1 signaling, but not a secondary event driven by a decline in lysosomal function.

## 3 DISCUSSION

The mechanism by which expanded GGGGCC/GGCCCC hexanucleotide repeats trigger neurotoxicity and cause ALS/FTD remains elusive. Our previous study, along with others, suggested that two arginine‐rich DPRs, poly‐GR and poly‐PR, can localize to the nucleolus and are most toxic in cells and animal models (Mizielinska et al., 2014; Tao et al., 2015), suggesting that RAN‐translated DPR toxicity contributes to disease pathogenesis of *C9orf72*‐linked ALS/FTD. Nevertheless, different disease mechanisms may act synergistically to cause C9orf72‐linked ALS. For example, although decreased expression of C9orf72 protein alone seems unlikely to cause disease, C9orf72 loss of function can be a disease modifier that helps to accelerate neurodegeneration. Of note, C9orf72‐mediated autophagy regulation may be important, since this protein quality control system is tightly linked to ALS/FTD and other neurodegenerative disorders (Ling et al., 2013).

The full autophagy process contains multiple steps, including (1) initiation by the ULK1/ATG13/FIP200/ATG101 complex, (2) phagophore nucleation by the PI3KC3 complex I, (3) phagophore expansion, (4) cargo sequestration, (5) membrane sealing, and (6) ultimate fusion between the autophagosome and the lysosome (Dikic & Elazar, 2018). Previous reports convincingly showed that C9orf72 can interact with the ULK1/ATG13/FIP200/ATG101 complex and regulate the initiation step of autophagy (Sellier et al., 2016; Sullivan et al., 2016; Ugolino et al., 2016; Webster et al., 2016; Yang et al., 2016). Interestingly, a very recent study showed that C9orf72 is able to interact with the autophagy receptor p62/SQSTM1, which plays a role in the cargo sequestration step of autophagy (Chitiprolu et al., 2018). In the present study, we find that C9orf72 can regulate global gene transcription of the autophagy–lysosome system through MITF/TFE3/TFEB, master regulators of lysosome biogenesis and autophagy (Figure 6). We show that C9orf72 affects mTORC1‐MITF/TFE3/TFEB signaling in a Rag GTPase‐dependent manner (Figures 3 and 4), suggesting that C9orf72 regulates the autophagy–lysosome pathway by affecting Rag GTPases (Figures 5 and 6). Our study, along with the above‐mentioned previous studies, shows that C9orf72 is an effective autophagy regulator that can play multiple roles in the regulation of autophagy. Importantly, a recent study showed that overexpression of C9orf72 may enhance autophagy initiation under normal condition, but may impair autophagy under stress conditions (Cali et al., 2019). Interestingly, we found that loss of C9orf72 (opposite to C9orf72 overexpression) might enhance neuronal autophagy under stress condition (Figure S7d). Given that under ALS/FTD disease condition, the neurons are under stress and therefore lack of C9orf72 may induce autophagy during the disease progress.

Taken together, the present study identifies Rag GTPases as a new interacting partner of C9orf72, and it provides mechanistic insights into C9orf72‐mediated regulation of mTORC1 signaling and its control of the autophagy–lysosome pathway. We and others have previously shown that mTORC1 activity is impaired in different ALS models, including TARDBP/TDP‐43 and VCP‐depleted cells and animals (Ching et al., 2013; Ying et al., 2016). In support of this notion, we found that mTORC1 activity was also decreased as a consequence of C9orf72 depletion (Figure 2), indicating that mTORC1‐mediated autophagy may be broadly altered in ALS disease pathogenesis. It is worth to note that although important connections between protective autophagy and ALS have been observed in genetic and preclinical studies, there are numbers of studies that suggest the treatment with general “autophagy inducers” such as rapamycin (mTOR inhibitor) does not help to ameliorate disease progression, or may even exacerbate neurotoxicity as seen in ALS animal models (Xia et al., 2016). This negative effect may be explained by the fact that rapamycin blocks mTOR activity and that functional mTOR signaling is potentially important for motor neuron homeostasis during ALS disease progression. Since C9orf72 hippodeficiency alone may not drive neurodegeneration, it will be interesting to explore the role of mTOR signaling in the background of toxic gain‐of‐function models, such as models with the expression of GGGGCC/GGCCCC RNA repeats and/or DPRs.

## 4 EXPERIMENTAL PROCEDURES

### 4.1 Plasmid constructs

Full‐length C9orf72 cDNA was first amplified by PCR using primers 5′‐GGAATTCCATGTCGACTCTTTGCCCAC‐3′ and 5′‐GCTCTAGAAAAAGTCATTAGAACATCTCGTT‐3′ from a human fetal brain cDNA library. Then, the PCR product was then inserted into the p3 × FLAG‐myc‐CMV‐24 vector at EcoRI/XbaI sites. C9orf72‐EGFP was generated by excising C9orf72 cDNA from FLAG‐C9orf72 and inserted it into pEGFP‐N3 vector at EcoR1/BamH1 sites. The deletion mutants of C9orf72 were generated by following primers: 5′‐ATGGGTGCTATAGATGTAAAG‐3′ and 5′‐GGATGAATTCGCGGCCGC‐3′ for FLAG‐C9orf72 Δ1–77; 5′‐ATGGCTTACTGGGACAATATT‐3′ and 5′‐GGATGAATTCGCGGCCGC‐3′ for C9orf72Δ1–30‐EGFP; 5′‐GATGGAAACTGGAATGGG‐3′ and 5′‐AGCACCACTCTCTGCATT‐3′ for C9orf72 Δ80–99‐EGFP; 5′‐GACAGCTGTCATGAAGGC‐3′ and 5′‐AGCACCACTCTCTGCATT‐3′ for FLAG‐C9orf72 Δ80–212; 5′‐GACAGCTGTCATGAAGGC‐3′ and 5′‐TTCTTCAGGAACACTGTG‐3′ for C9orf72 Δ199–212‐EGFP; 5′‐GAACATATTTATAATCAG‐3′ and 5′‐AATATCATCATCATTGAG‐3′ for FLAG‐C9orf72 Δ212–322 (ΔDENN); 5′‐AAAGCCTTGACACTAATA‐3′ and 5′‐ACAGGGTGGCATCTGCTT‐3′ for FLAG‐C9orf72 Δ322–407; 5′‐GGATCCGAACAAAAACTC‐3′ and 5′‐ACAGGGTGGCATCTGCTT‐3′ for FLAG‐C9orf72 Δ322–481; and 5′‐GCAGAGGGCGATCTTAAC‐3′ and 5′‐GTGAAGGACAAGTAGAAA‐3′ for C9orf72 Δ407–440‐EGFP. LAMP1‐RFP, HA‐raptor, pRK5‐HA GST Rag A^GTP^ (Rag A Q66L), pRK5‐HA GST Rag C^GDP^ (Rag C S75L), FLAG‐TFEB, and TFEB‐EGFP plasmids were described previously (Xia et al., 2016). The autophagic flux reporter pMRX‐IP‐GFP‐LC3‐RFP‐LC3ΔG plasmid was a kind gift from Dr. Noboru Mizushima: Addgene plasmid # 84572. The following plasmids were gifts from Dr. David Sabatini: Addgene plasmid # 19299, pRK5‐HA GST Rag A^GDP^ (Rag A T21L); Addgene plasmid # 19306, pRK5‐HA GST Rag C^GTP^ (Rag C Q120L). All of the constructs were confirmed by sequencing.

### 4.2 siRNAs

A nontargeting oligonucleotide was used as a negative control. The following small interfering RNAs were ordered from QIAGEN: siRNA targeting human C9orf72 (si‐C9orf72, SI04190193); human C9orf72 (targeting noncoding region, SI04360755), and mouse C9orf72 (SI00831145). The following siRNAs were ordered from GenePharma: human C9orf72‐2 (si‐C9orf72‐2): 5′‐GUCCUAGAGUAAGGCACAUTT‐3′; human Rag A: 5′‐CCAACUUCGCUGCUUUCAUTT‐3′; human Rag B: 5′‐GGACAUGCACUAUUACCAAUTT‐3′; human raptor: 5′‐UGGCUAGUCUGUUUCGAAATT‐3′; MITF: 5′‐CGGGAAACUUGAUUGAUCUUUTT‐3′; human TFE3: 5′‐GCAGCUCCGAAUUCAGGAACUTT‐3′; and human TFEB: 5′‐GAGACGAAGGUUCAACAUCAATT‐3′. siRNAs targeting Rag A and Rag B were co‐transfected to block the expression of Rag proteins (si‐Rags).

### 4.3 Cell culture, transfection, and drug treatments

HeLa, human embryonic kidney 293 cells (HEK 293), human embryonic kidney 293T cells (HEK 293T), and mouse motor neuron cells (NSC‐34) were cultured in Dulbecco's modified Eagle's medium (DMEM) (Gibco) containing 10% fetal bovine serum (FBS) (Gibco) with penicillin (100 U/ml) and streptomycin (0.1 mg/ml), and were maintained at 37°C and a humidified 5% CO_2_ atmosphere. Primary culture of cortical neurons was described before (Xia et al., 2015). Cells were transfected with siRNAs using the RNAi MAX reagent (Invitrogen) upon splitting in Opti‐MEM and transfected with plasmids by Lipofectamine 2000 transfection reagent (Invitrogen) at 90% confluence according to the manufacturer's instructions. Cells were starved for 1 hr by incubating in Earle's balanced salt solution (EBSS) (Gibco) and/or re‐fed with full culture medium for indicating time. Cells were treated with Torin‐1 (250 nM) (Tocris Bioscience) for 1 hr. For LysoTracker staining, HEK 293 cells were stained with 100 nM LysoTracker Red DND‐99 (Invitrogen) for 30 min, and then, the quantification of LysoTracker fluorescence intensity was performed. DAPI and Hoechst (Sigma) were used to stain the nucleus. For lysosomal PH measurement, control and C9orf72‐depleted cells were loaded with 1 mg/ml LysoSensor Yellow/Blue dextran (Thermo Fisher Scientific) for 12 hr. Then, the cells were subjected to lysosomal pH quantification as previously reported (Yanagawa et al., 2007).

### 4.4 Antibodies

The following primary antibodies were used in our experiments: anti‐GAPDH antibody (Proteintech), anti‐Histone H3 antibody (Proteintech), anti‐phospho‐p70S6K antibody (T389) (Cell Signaling Technology), anti‐p70S6K antibody (Epitomics), anti‐LAMP1 antibody (Abcam), anti‐LAMP2 antibody (Santa Cruz), anti‐TFEB antibody (Cell Signaling Technology), anti‐p62 antibody (Enzo Life Sciences), anti‐mTOR antibody (Cell Signaling Technology), anti‐HA antibody (Santa Cruz), anti‐FLAG antibody (Sigma), anti‐GFP antibody (Santa Cruz), anti‐Rag B antibody (Cell Signaling Technology), anti‐raptor antibody (Cell Signaling Technology), anti‐α‐Tubulin (Proteintech), and anti‐C9orf72 (Santa Cruz). The following secondary antibodies were used: horseradish peroxidase‐conjugated sheep anti‐mouse and anti‐rabbit antibodies (Jackson ImmunoResearch Laboratories). The following fluorescent secondary antibodies were used: Alexa Fluor 594‐conjugated goat anti‐rabbit IgG (Proteintech), Alexa Fluor 594‐conjugated goat anti‐mouse IgG (Proteintech), Alexa Fluor 488‐conjugated goat anti‐rabbit IgG (Proteintech), Alexa Fluor 488‐conjugated Goat anti‐mouse IgG (Proteintech), Alexa Fluor 660‐conjugated goat anti‐mouse IgG (H + L) highly cross‐adsorbed antibody (Invitrogen), and Alexa Fluor 405‐conjugated goat anti‐rabbit IgG (H + L) secondary antibody (Invitrogen).

### 4.5 Immunoblot

Cells were lysed in cell lysis buffer containing 50 mM Tris‐HCl (pH 7.6), 150 mM NaCl, 0.5% sodium deoxycholate, 1% Nonidet P‐40 (NP‐40), and protease inhibitor cocktail (Roche). Proteins were separated by SDS‐PAGE (polyacrylamide gel electrophoresis) and transferred onto a PVDF membrane (polyvinylidene difluoride membrane; Millipore). Immunoblot analysis was standardly performed as previously reported (Tao et al., 2015). The proteins were visualized with an ECL detection kit (Thermo Fisher Scientific). Visualized digital immunoblot images were obtained using ChemiScope 3300 Mini (CLiNX). During capture of the images, the band detection was within the linear range.

### 4.6 GST pull‐down and Immunoprecipitation

HEK 293T cells expressing HA‐glutathione S‐transferase (GST) Rag‐fused proteins were lysed, and the supernatants were incubated with G4B magnetic beads (GSH Magnetic Beads for GST Tag Protein Purification, Selleck) for 4 hr at 4°C. After washing three times with precold PBS, bound proteins were eluted from the beads by SDS sample buffer and subjected to immunoblot analysis. For immunoprecipitation assay, the cell lysates were centrifuged at 4°C at 13,523 *g* for 30 min. The supernatants were subjected to immunoprecipitation with FLAG antibody coupled with protein G Sepharose (Roche) at 4°C for 4 hr. After washing three times with cell lysis buffer, bound proteins were eluted with SDS sample buffer for immunoblot analysis.

### 4.7 Subcellular fractionation

To obtain nuclear and cytoplasmic fractions, cells were harvested, washed with ice‐cold PBS, and re‐suspended with ice‐cold sucrose buffer (10 mM sucrose, 1 mM CaCl2, 10 mM MgAc, 2.5 mM EDTA, 1 mM DTT, 1 mM PMSF, and 0.5% NP‐40). Then, the cells were then incubated in sucrose buffer for 20 min followed by separation into cytoplasm (supernatants) and nucleus (pellet) by centrifugation at 600 *g* for 15 min at 4°C. The pellet was washed twice using sucrose buffer without NP‐40 and finally re‐suspended in cell lysis buffer. For NP‐40 soluble and insoluble fractionation experiments, cells were first lysed in cell lysis buffer and then centrifuged at 12,000 *g* at 4°C or 30 min. The supernatants were collected as NP‐40 soluble parts. NP‐40 insoluble pellets were dissolved in pellet buffer (1% SDS, 1% NP‐40).

### 4.8 Immunofluorescence

HEK 293 cells were grown on 24‐well glass bottom cell imaging plate (Eppendorf), washed with PBS, and fixed with 4% paraformaldehyde in PBS at room temperature for 10 min. Then, the cells were permeabilized and blocked with 0.1% Triton X‐100 combined with 0.2% FBS in PBS for 10 min. Alternatively, cells were permeabilized with 0.1% saponin (Sigma) in PBS at room temperature for 20 min. Cells were incubated with primary antibodies diluted with PBST at room temperature for 4 hr, washed gently with PBST for 3 times, and then incubated with 5 µg/ml above‐mentioned fluorescent secondary antibodies at room temperature for 2 hr. 4′6‐Diamidino‐2‐phenylindole (DAPI) (Sigma) was used to stain nucleus for 5 min. The stained cells were visualized using Nikon (Wu et al., 2012) or Zeiss LSM710 confocal microscope (Fang et al., 2017; Ren, Zhao, Cao, & Zhen, 2016).

### 4.9 Quantitative real‐time PCR

Total RNA from cells was extracted using TRIzol Reagent (Invitrogen) as previously described (Yang et al., 2018). The RNA was reverse‐transcribed into cDNA using PrimeScript RT Master Mix (Takara). Real‐time PCR analysis was carried out for quantitative measurement of the abundance of target RNA with SYBR Green Real‐Time PCR Master Mix (Takara) in a PCR detection system (Applied Biosystems). The following ALS/FTD gene‐related primers were used: human UBQLN2: 5′‐CATAGGACCCACTGGCCCTG‐3′ and 5′‐GCTGAATGAACTGCTGGTTGGG‐3′, human PINK1: 5′‐AGACGCTTGCAGGGCTTTC‐3′ and 5′‐GGCAATGTAGGCATGGTGG‐3′, human VCP: 5′‐CAAACAGAAGAACCGTCCCAA‐3′ and 5′‐TCACCTCGGAACAACTGCAAT‐3′, human p62: 5′‐AAGCCGGGTGGGAATGTTG‐3′ and 5′‐CCTGAACAGTTATCCGACTCCAT‐3′, and human OPTN: 5′‐AGCAAACCATTGCCAAGC‐3′ and 5′‐TTTCAGCATGAAAATCAGAACAG‐3′. All the other primers used in this paper were previously described (Xia et al., 2016).

### 4.10 Electron microscopy (EM)

Cells were washed, centrifuged, and followed by addition of a drop of glutaraldehyde to the cell suspension. Then, the cells were fixed in suspension with 0.2 M Na‐phosphate buffer (pH 7.4) containing 2.5% glutaraldehyde at 4°C for 2 hr. Then, the samples were embedded and subjected to EM observations.

### 4.11 Statistical analysis

Immunoblot densitometric analyses from three independent experiments were performed using software Photoshop 7.0 (Adobe). Lysosome fluorescence intensity analyses of the LysoTracker staining were performed using the ImageJ software (National Institutes of Health). EGFP‐fused TFEB, TFE3, and MITF nuclear localization was determined by visual inspection. The obtained data were used to generate charts using Prism 6.0 (GraphPad Software). *p*‐values were obtained as indicated in figure legends. To determine the mTOR and lysosome (LAMP1 positive) colocalization, we used Fiji v.1.52p software (ImageJ). Individual channels were segmented first, and the vesicles were identified with binary mask via the intensity threshold function. Manders' colocalization coefficients were measured using Coloc 2 colocalization function. The mean value of colocalization data from 10 fields for each group was normalized to the control group.

## CONFLICT OF INTEREST

The authors declare that they have no conflicts of interest with the contents of this article.

## AUTHOR CONTRIBUTIONS

M.W., H.W., G.W., and Z.Y. designed research; M.W., H.W., Z.T., Q.X., Z.H., and Z.Y. performed experiments; J.P., X.Z., G.W., and Z.Y. provided advice and analyzed data; and M.W., H.W., J.P., and Z.Y. wrote and edited the manuscript.

## Supporting information

FigS1‐S7

## Data Availability

The data that support the findings of this study are available from the corresponding author upon reasonable request.
