# Supplementary material for: C9orf72 associates with inactive Rag GTPases and regulates mTORC1‐mediated autophagosomal and lysosomal biogenesis
Source: Aging Cell. 2020 Feb 25;19(4):e13126. doi: 10.1111/acel.13126 (PMC7189992; doi:10.1111/acel.13126)
Supplement: Supplementary file 1 — FigS1‐S7 [file ACEL-19-e13126-s001.pdf]

Supplementary Figures

Figure S1. (a) HEK 293 cells were transfected with indicated siRNAs. Seventy-two hours after transfection, the protein levels were detected by immunoblot analysis using indicated antibodies. (b) Quantification of co-localization of mTOR and LAMP1-RFP (indicating lysosome) in Figure 3a were made from 10 fields in 3 trials for each group. Data were normalized to control group and presented as mean ± S.E.M., ns, not significantly different; **, *P*<0.01, one-way ANOVA.

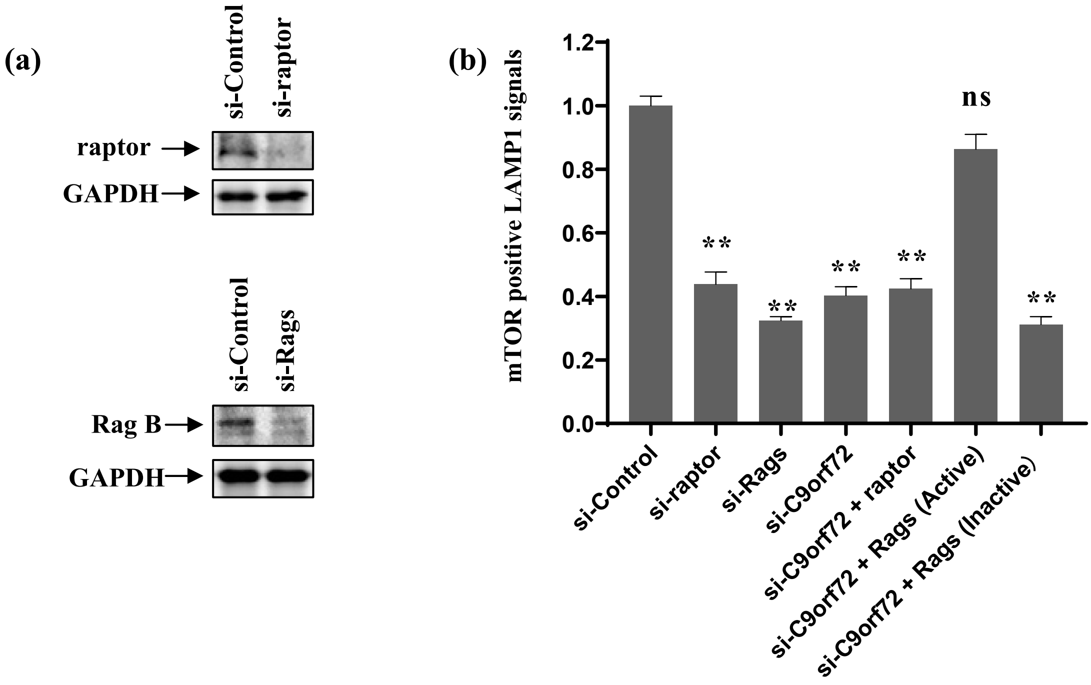

Figure S2. (a) Primary cultured cortical neurons from E17 mouse were transfected with control or mouse C9orf72 siRNA on DIV 11. Then the cells were subjected to immunofluorescence staining using LAMP2 (red) and TFEB (green) antibodies. DAPI (blue) was used for nuclear staining. Arrows indicate the nucleus. Scale bars, 10 µm. (b) HEK 293 cells were transfected with indicated siRNAs for 48 h, followed by transfection of TFEB-EGFP, LAMP1-RFP and HA or HA-GST-Rags (Inactive) for another 24 h. Then the cells were fixed and stained with DAPI before being observed under confocal microscopy. Scale bar, 5 µm. (c) The quantitative data from three independent experiments in (a) were shown as means ± S.E.M, ns, not significantly different; **, *P*<0.01, one-way ANOVA. (d) HEK 293 cells were transfected with the indicated siRNAs. After 48 h, the cells were transfected with TFEB-EGFP (green), along with HA Tag (mock), HA-raptor or HA-GST-Rags for another 24 h. The cells were fixed and observed under microscope. Scale bars, 5 µm.

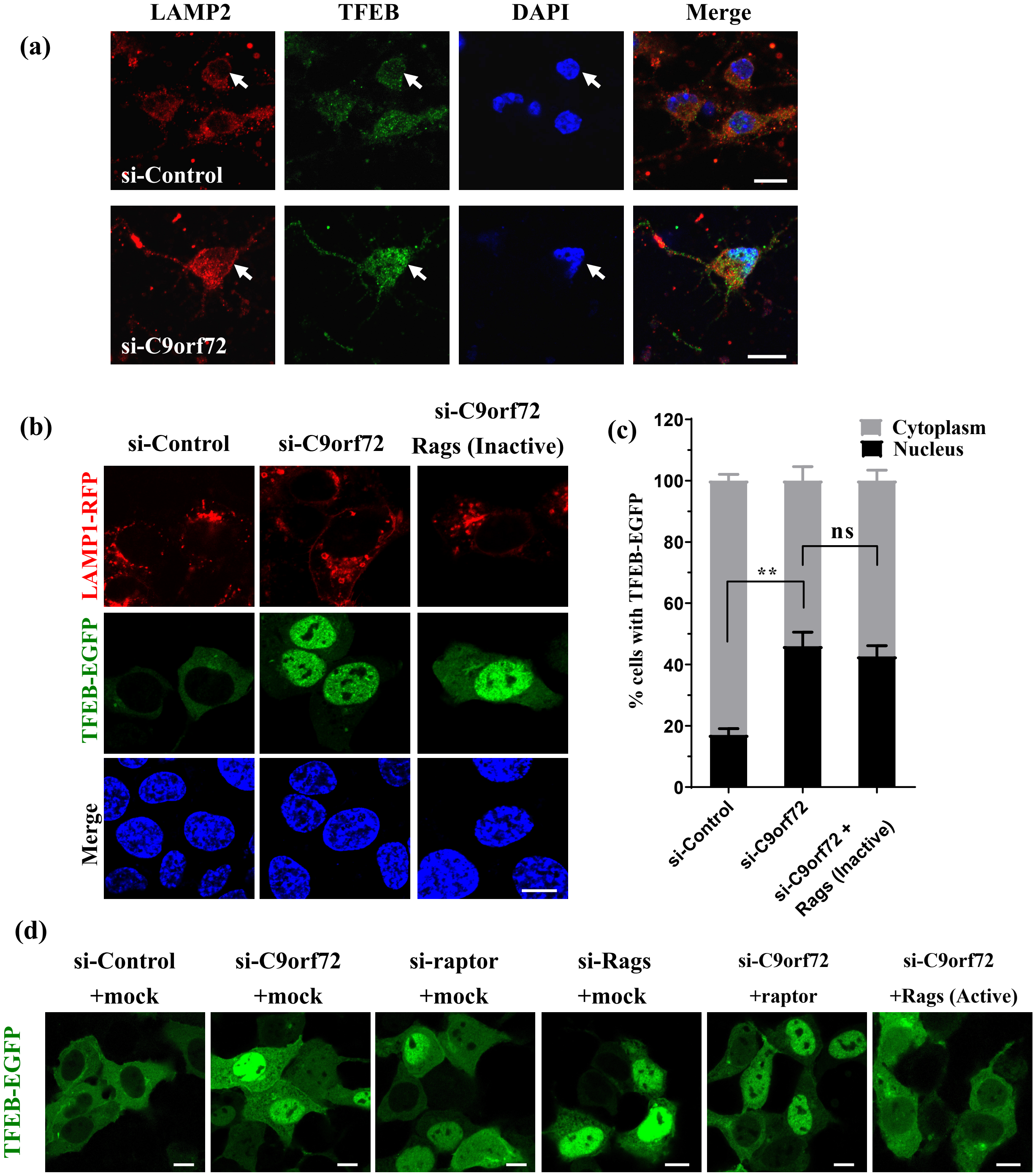

Figure S3. Neuronal NSC-34 cells were transfected with mock vector, HA-GST-tagged constitutively inactive or active Rags. Then the cell supernatants of cell lysates were subjected to GST pull down assay. Bound endogenous C9orf72 protein was detected with antibody against C9orf72.

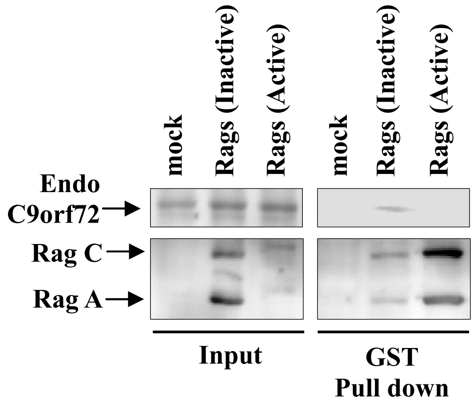

Figure S4. C9orf72 colocalizes with Rag GTPases through its DENN domain. FLAG-tagged full length or truncated C9orf72 and HA-GST-tagged inactive Rags were co-transfected into HEK 293 cells. Twenty-four hours later, the cells were fixed and subjected to immunofluorescence staining using FLAG and HA antibodies. Scale bars, 5 µm.

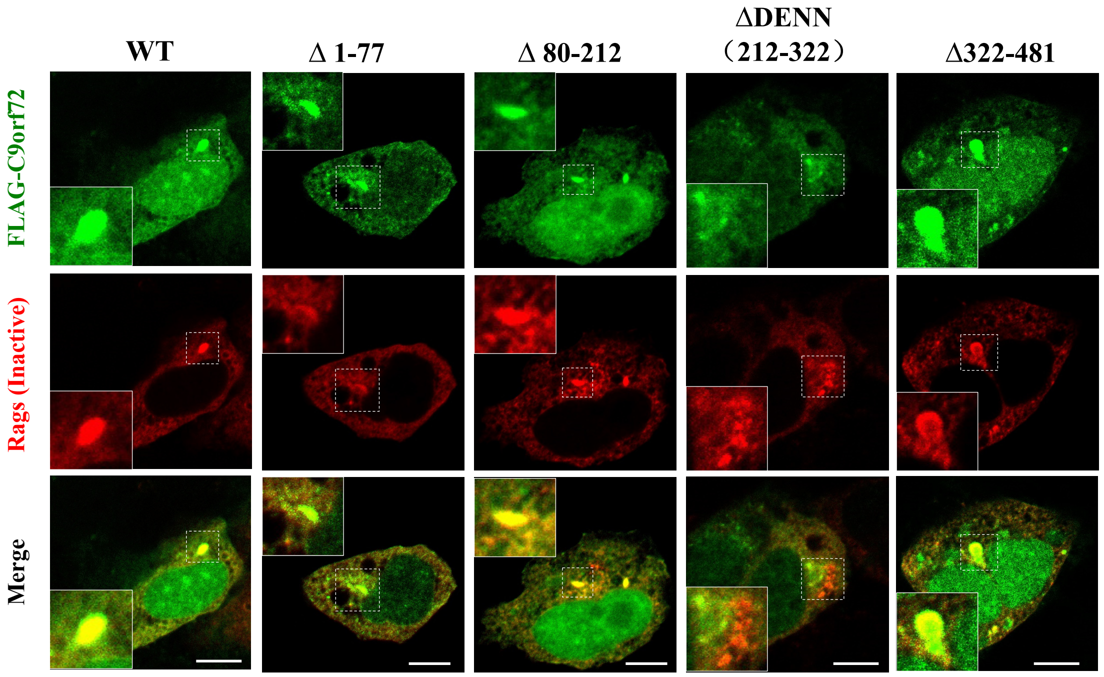

Figure S5. (a) HEK 293 cells were transfected with indicated siRNAs for 48 h, followed by transfection with FLAG-C9orf72 or FLAG-C9orf72 lacking DENN domain (FLAG-C9orf72 ΔDENN) for another 24 h. Then the cells were stained with Lysotracker for lysosomes and Hoechst for nucleus, respectively. (b) HEK 293 cells were co-transfected with si-Control or si-C9orf72 (targeting non-coding region) siRNAs. Forty-eight hours later, the cells were transfected with TFEB-EGFP and LAMP1-RFP, along with FLAG-tagged wild type C9orf72 or C9orf72 ΔDENN. Another 24 h later, the EGFP or RFP fused proteins were visualized using confocal microscopy. Scale bars, 5 μm.

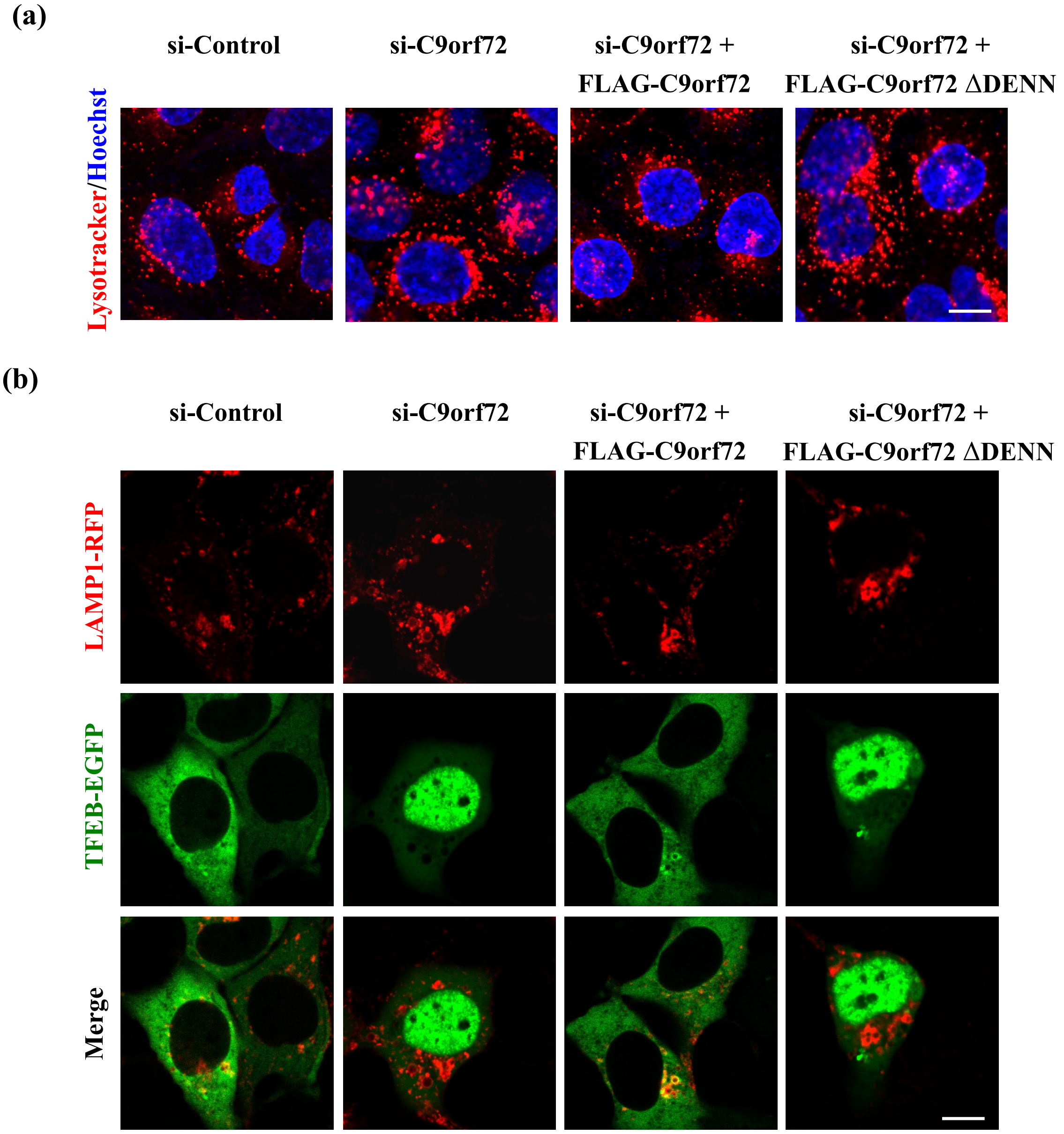

Figure S6. (a and b), HEK 293 cells were transfected with control or C9orf72 siRNAs. Seventy-two hours later, the cells were processed for qRT-PCR analysis. The mRNA levels of ALS-associated genes *TARDBP*, *UBQLN2*, *OPTN*, *VCP* and *SQSTM1*/*p62* (a) as well as autophagic genes *ATG5*, *ATG16L1*, *ATG9B* and *UVRAG* (b) were quantified. The relative data were normalized to GAPDH levels. Data were presented as means ± S.E.M., ns, not significantly different; *, P<0.05; **, P<0.01, one-way ANOVA.

(a)

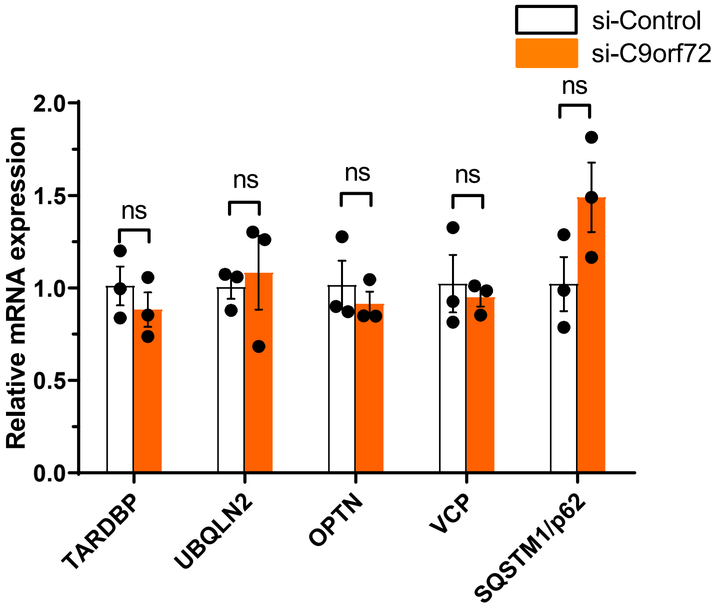

(b)

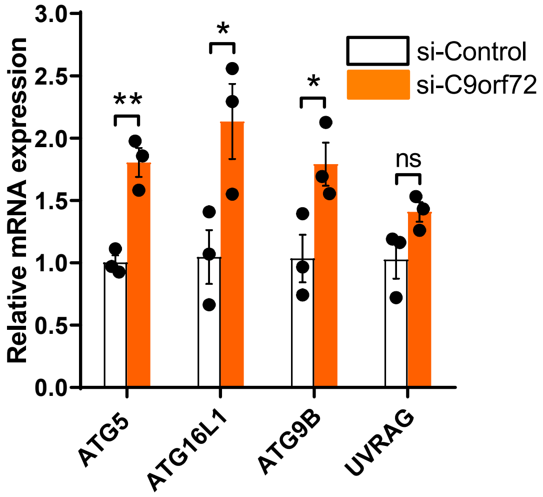

Figure S7. (a) HEK 293 cells were first transfected with indicated siRNAs. Forty-eight hours later, the cells were re-transfected with GFP-LC3-RFP-LC3ΔG. After 24 h, the cells were collected for immunoblot analysis. (b) Similar experiment was performed as in a. After 12 h Bafilomycin A1 treatment, the cells were fixed and visualized using confocal microscopy. Scale bar, 5 μm. (c) HEK 293 cells were transfected with the indicated siRNAs. After 48 h, the cells were transfected with FLAG, FLAG-C9orf72 or FLAG-C9orf72 ΔDENN for another 24 h. The cells were lysed and subjected to immunoblot with indicated antibodies. (d) Primary cultured cortical neurons from E17 mouse were transfected with either control or C9orf72 siRNA on DIV 11. Seventy-two hours later, the neurons were incubated with EBSS for 1 h to better induce neuronal autophagy, and the cell lysates were subjected to immunoblot with indicated antibodies. (e) Lysosomal pH was quantified ratio-metrically using LysoSensor Yellow/Blue dextran reporter. HEK 293 cells transfected with si-Control or si-C9orf72 siRNA were loaded with 1 mg/mL LysoSensor Yellow/Blue dextran for 12 h then subjected to lysosomal pH measurement. Data were presented as means ± S.E.M., ns, not significantly different, one-way ANOVA.

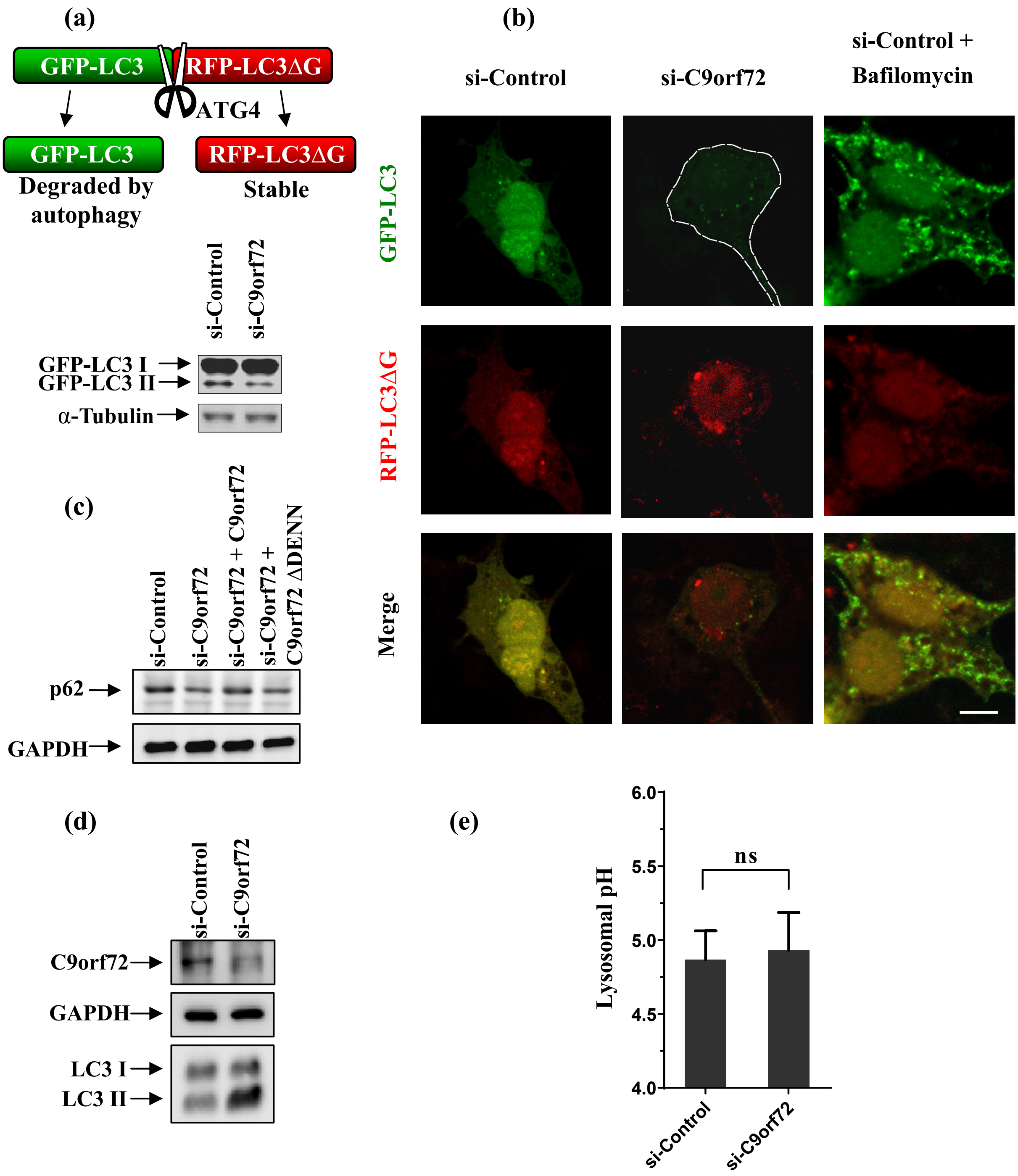

(a)
GFP-LC3
RFP-LC3ΔG
ATG4
GFP-LC3
RFP-LC3ΔG
Degraded by autophagy
Stable
si-Control
si-C9orf72
GFP-LC3 I
GFP-LC3 II
α-Tubulin
(b)
si-Control
si-C9orf72
si-Control + Bafilomycin
GFP-LC3
RFP-LC3ΔG
Merge
(c)
si-Control
si-C9orf72
si-C9orf72 + C9orf72
si-C9orf72 + C9orf72 ΔDENN
p62
GAPDH
(d)
si-Control
si-C9orf72
C9orf72
GAPDH
LC3 I
LC3 II
(e)
Lysosomal pH
6.0
5.5
5.0
4.5
4.0
ns
si-Control
si-C9orf72
